# Peritoneal Tuberculosis during Infliximab Treatment in a Patient with Ulcerative Colitis Despite a Negative Quantiferon Test

**DOI:** 10.3390/pathogens10050535

**Published:** 2021-04-29

**Authors:** Anna Colombo, Mauro Giuffrè, Lory Saveria Crocè, Sergio Venturini, Renato Sablich

**Affiliations:** 1 Department of Medicine, Surgery and Health Sciences, University of Trieste, 34149 Trieste, Italy; anna.colombo@studenti.units.it (A.C.); lcroce@units.it (L.S.C.); 2 Department of Infectious Diseases, Azienda Sanitaria Friuli Occidentale, Pordenone Hospital, 33170 Pordenone, Italy; sergio.venturini@asfo.sanita.fvg.it; 3 IBD Unit, Department of Gastroenterology, Azienda Sanitaria Friuli Occidentale, Pordenone Hospital, 33170 Pordenone, Italy; renato.sablich@asfo.sanita.fvg.it

**Keywords:** peritoneal tuberculosis, inflammatory bowel disease, Infliximab, Quantiferon

## Abstract

Infliximab is an IgG1 antitumor necrosis factor monoclonal antibody that is commonly used to treat inflammatory bowel disease (IBD) and other autoimmune disorders. However, it is known to increase the risk of reactivation of latent tuberculosis (LTBI) due to its capability to disrupt TB granulomas. We describe a case of extrapulmonary TB in a patient with ulcerative colitis who was treated with Infliximab after a negative Quantiferon Test. In addition, we report briefly on the current controversy about the appropriateness, interval, and methods for the repeated screening of latent TB in IBD patients that are treated with antitumor necrosis factor alpha (TNF-α) antibodies.

## 1. Introduction

Infliximab is a chimeric monoclonal antibody that targets tumor necrosis factor alpha (TNF-α), a pro-inflammatory cytokine that plays a key role in inflammatory bowel disease (IBD) [1]. Anti-TNF-α agents have proven effective in treating several immune-mediated conditions. Their availability since the early 2000s has increased the chances of success in achieving clinical remission, mucosal healing, and improved quality of life in patients with moderate-to-severe IBD [2]. However, they increase the risk of opportunistic infection and, in particular, the reactivation of latent tuberculosis infection (LTBI) by interfering with the physiological role of TNF-α in inducing intracellular microorganism phagocytosis and promoting the development of granulomas [3]. In the absence of TNF-α, granulomas can dissolve and release the (Koch’s Bacillus) BK [4]. Consequently, screening for LTBI has become mandatory before anti-TNF-α therapy and includes a combination of the patients’ history, chest radiography, tuberculin skin test (TST), and/or interferon-gamma release assays (IGRAs) [5]. We report an uncommon case of extrapulmonary tuberculosis (TB) in a patient that was treated with Infliximab and without previous evidence of latent infection. 

## 2. Case Report

**Figure 1:**
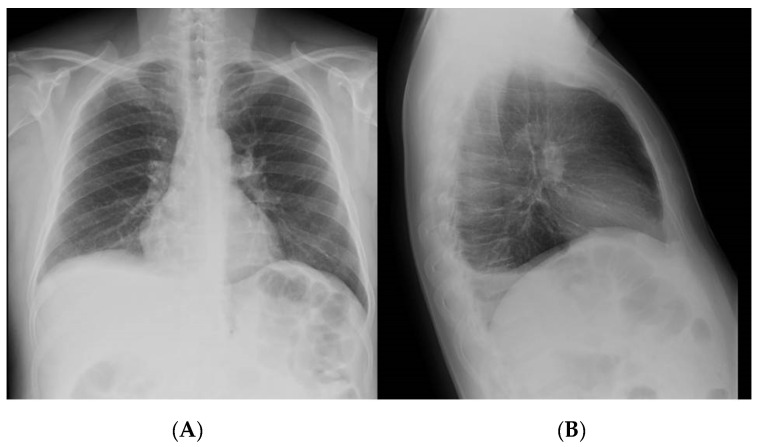
Chest X-ray—right posterior basal pleural effusion as shown in anteroposterior (**A**) and lateral (**B**) views.

**Figure 2:**
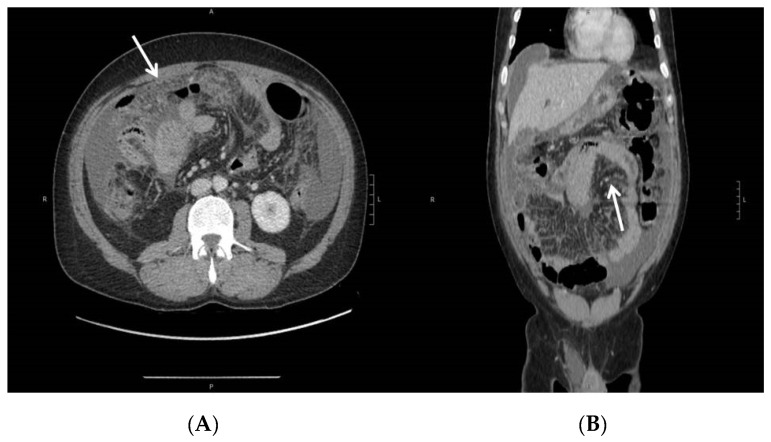
Abdominal CT—peritoneal effusion, diffuse suffusion of mesenteric adipose tissue, and thickening of the parietal peritoneum as shown in axial (**A**) and coronal (**B**) views. Diffuse parietal thickening (arrows) of the ileo-jejunal loops, with preserved parietal enhancement.

**Figure 3:**
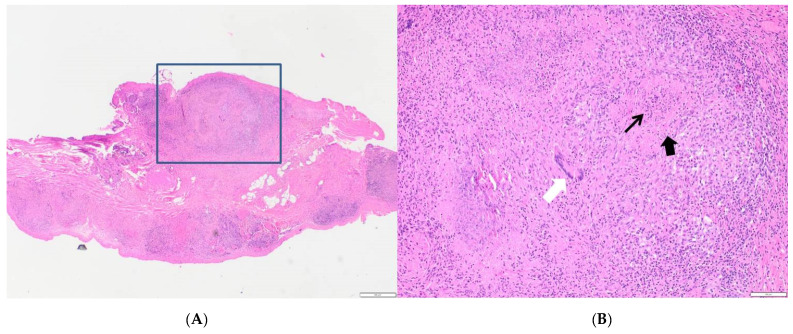
(**A**) Peritoneal biopsy: multinodular inflammatory lesions (E&E, 2×). (**B**) Selected area: central slightly eosinophilic and granular necrotic area (arrow) surrounded by large pink epithelioid cells and fibroblasts (arrowhead), small lymphocytes, and isolated multinucleate giant cells of Langhan’s type (white arrow) (E&E, 10×).

A 48-year-old male with a history of steroid-dependent ulcerative colitis (UC) since 2017 was admitted to the Department of Gastroenterology of Pordenone Hospital in Northeast Italy in December 2018 due to a high intermittent fever, abdominal pain, and diarrhea, without a productive cough, dyspnea, sweating, or symptoms of urinary tract infections. The patient had immigrated from Morocco in 2007, received four scheduled 10 mg/kg infusions of biosimilar Infliximab CT-P13 (Inflectra^®^—Celltrion Healthcare, Co., Ltd., Incheon, Korea) over the last five months following a negative Quantiferon test (QFT^®^-Plus, Qiagen, Germany) [6]. Physical examination revealed a tense abdomen with tenderness in the right quadrants, but no sign of acute peritonitis. A colonoscopy diagnosed a Mayo 1 extended colitis and normal mucosa in the terminal ileum. A laboratory workup showed elevated C-reactive protein (23 mg/dL), thrombocytosis (781,000/mm^3^), hyperferritinemia (866 µg/L), and hypoalbuminemia (2.7 g/dL), while procalcitonin, electrolytes, liver, and kidney function were normal. Cytology, serology, and genetic stool testing excluded malaria, leishmaniasis, bacterial, fungal, CMV, and any other viral infection. An HIV Ag/Ab Combo test (Advia Centaur XP, Siemens Healthcare Diagnostics, Inc., Erlangen, Germany) produced a negative result. A chest X-ray revealed a right pleural effusion (Figure 1). The abdominal ultrasound showed the presence of ascites with a thickened mesentery, but no signs of active inflammatory disease in the small or large bowel. Abdominal computed tomography (CT) confirmed the ultrasound findings and showed diffuse retroperitoneal non-calcific lymphadenopathy (Figure 2). Chest CT showed pleural effusion with localized thickening of the visceral pleura and slightly enlarged diffuse mediastianal lymphnodes without any rim sign. In contrast, no lesion of the pulmonary parenchyma was detected. Following the insertion of thoracic drainage, pleural effusion revealed exudate hallmarks (1258 leucocytes/µL, 4.8 g/dL proteins, 100 mg/dL glucose, 286 U/L LDH) in the absence of malignant cells and acid-fast bacilli. On paracentesis, the ascitic fluid was found to be turbid, with 760 leucocytes/µL (12% neutrophils), 176 mg/dL glucose, 4.6 g/dL proteins, and negative standard cultural and cytologic examinations. Fever, elevated CRP without evidence of infected sites, failure of conventional antibiotic treatment (piperacillin/tazobactam and levofloxacin), recent exposure to anti-TNF-α, and ethnicity of the patient raised suspicion of peritoneal TB. At laparoscopy, the peritoneum and omentum appeared disseminated with small whitish nodules containing typical TB granulomas at histology (Figure 3). A repeated Quantiferon test was found to be indeterminate because of the high reactivity of the negative control, with the positive control (mitogen tube) >10 UI/mL. According to guidelines [7], a standard anti-TB regimen with four drugs was empirically started (“intensive phase”) with isoniazid 300 mg, rifampicin 600 mg, pyrazinamide 1500 mg, and ethambutol 1200 mg, which was set based on the patient’s weight, and each were given once a day for 13 weeks, followed by isoniazid 300 mg and rifampicin 600 mg (“continuation phase”) for an additional seven months. Fully susceptible *M. tuberculosis* was finally isolated using a culture from peritoneal fluid, confirming the diagnosis of peritoneal TB. A small amount of sediment from peritoneal fluid was used to prepare smears for Ziehl–Neelsen staining. The results of the peritoneal fluid sediment were negative, probably due to a low number of bacilli per milliliter, and was in agreement with the sensitivity of the direct acid-fast smear examination, which was lower than that of the culture methods. Concentrated samples for inoculation were prepared using the NaCl–NaOH decontamination method [8]. The same amount of each concentrated sample was inoculated into vials of the BACTEC MGIT System (Becton, Dickinson and Company, Franklin Lakes, NJ, USA). A 0.25 mL amount of concentrated sample was inoculated onto a Lowenstein–Jensen slant. The Lowenstein–Jensen slant was incubated at 37 °C and inspected weekly for growth over an 8-week period. BACTEC MGIT vials were monitored continuously by the BACTEC MGIT System. The growth of mycobacteria was verified using microscopy (Ziehl–Neelsen staining). Matrix-assisted laser desorption ionization time-of-flight (MALDI-TOF) mass spectrometry (MS) was used to identify *M. tuberculosis*. Indirect susceptibility testing was performed using the BACTEC MGIT System. The BACTEC MGIT System was positive on the 23rd day and the Lowenstein–Jensen slant culture was positive on the 36th day. Streptomycin, isoniazid, rifampin, and ethambutol were tested as primary agents: no resistances were found. The absence of pulmonary parenchymal involvement, together with the absence of epidemiological criteria, largely excluded a primary TB. 

The patient’s clinical condition slowly improved and he left the hospital after 45 days. Fatigue, mild fever, and elevated inflammatory markers persisted for months. An abdominal CT in April 2019 showed omental thickening with moderate contrast enhancement, and sub-glissonian, perisplenic, and periumbilical abscesses, which were drained percutaneously. The specimens tested negative for BK and the patient was judged healed in October 2019 despite unvaried CT findings. A repeated colonoscopy showed extensive Mayo 2 colitis and the patient was put on mesalazine 3.2 g a day, unexpectedly achieving clinical remission in two weeks.

## 3. Discussion

Although TNF-α antagonists have clearly contributed to improving the outcome of chronic inflammatory diseases, interference with the host’s immune defenses is still an issue, particularly regarding the risk of LTBI reactivation. Currently, one-fourth of the world’s population is estimated to carry LTBI, with the majority of individuals being asymptomatic [9]. In 2017, 6.7 million incident cases were reported worldwide, with high variability in TB incidence among different countries. The incidence in Morocco is more than ten times higher than in Italy (99/100,000 vs. 6.9/100,000 population per year) [10]. In patients treated with anti-TNF-α, TB is mainly due to the reactivation of a latent infection and sometimes presents with extrapulmonary involvement. Approximately 15% of cases of reactivation occur at extrapulmonary sites without active pulmonary TB [11]. Peritoneal tuberculosis is particularly subtle, difficult to diagnose, and hard to treat. Cross-sectional imaging may drive diagnostic suspicion and help with staging, but paracentesis is still considered a major diagnostic tool [12]. Unfortunately, the microscopic detection of *M. tuberculosis* in the ascitic fluid occurs in less than 5% of cases, while culturing takes 6 to 8 weeks, with a positivity rate ranging from 20% to 83% [13]. Therefore, laparoscopy, although invasive, very often becomes necessary [12], emphasizing the need for the accurate screening of TB before using any anti-TNF-α monoclonal antibody [14]. The screening tests based on interferon-gamma release assays (IGRAs) have higher sensitivity and specificity than TST in immunosuppressed subjects. IGRAs include an enzyme-linked immunosorbent assay (ELISA-QuantiFERON-TB, which was used in our case) and an enzyme-linked immunospot assay (ELISpot-TSPOT.TB) that measures the IFN-γ concentration (ELISA) or IFN-γ-secreting T cells (ELISpot) in response to antigens expressed by *M. tuberculosis* [15]. It is difficult to establish the real sensitivity and specificity of the two IGRAs because of the absence of a gold standard for diagnosing LTBI. However, despite a pooled specificity of 97–98% and a sensitivity of 93–95% for the assay we used, false-negative results may still occur in different conditions [16], such as sampling before the development of a cellular immune response, the presence of comorbid conditions, the use of drugs affecting the immune response, and errors during the analytical or preanalytical phase [6,17]. Our patient received corticosteroid therapy before the first Quantiferon test (QTF). There is a concern that IGRA may not be sensitive enough in patients on anti-TNF-α. Furthermore, a negative impact on IGRA results was also reported with other immunosuppressive agents, including steroids or thiopurines. This effect appeared to be more critical in the QFT than the T-SPOT test, although there were fewer studies that assessed T-SPOT, resulting in lower statistical power [18]. Some studies reported a higher sensitivity with less indeterminate results with the T-SPOT test compared to the QFT [19]. Likewise, the test result may be indeterminate, suggesting the need to repeat screening during follow-up. Rescreening during biological therapy is not mandatory according to the Centers for Disease Control and Prevention, unless patients are at risk for TB [14], and the British Society of Gastroenterology recommends TB screening only before starting anti-TNF-α [20]. However, studies in rheumatologic patients with a previous negative QTF report a delayed positivization in 12.5 to 29% of the cases [21,22] and the American College of Rheumatology currently recommends annual testing [23]. According to a recent cohort study published by Abitbol et al. [24], who described the incidence of TB in 44 IBD patients undergoing anti-TNF treatment despite a negative screening test, extrapulmonary involvement was detected in 91% of cases, with peritoneal involvement in 15%. More case reports that describe the clinical characteristics of IBD patients developing peritoneal TB despite a negative screening test during anti-TNF therapy are summarized in Table 1. Interestingly, our patient, as well as two-thirds of the reported cases, were on immunosuppressant therapy at the time of LTBI screening. Furthermore, in one case, the screening was based on Quantiferon, as in our patient. The diagnosis was made with the isolation of *M. tuberculosis* in two cases, while in a third, anti-TB therapy was initiated on the basis of high levels of adenosine deaminase (ADA) in ascitic fluid, which, unfortunately, was not available in our patient.

The present report reinforces the need for gastroenterologists to screen patients for LTBI before any immunosuppressant therapy (including corticosteroids) and repeat TB test in IBD patients if they are classified as being at high risk of being carriers. The use of TB-SPOT as an alternative to Quantiferon might offer some advantages in this setting. Furthermore, the choice of other classes of biologics, such as anti-interleukin or anti-integrins, which have no substantial risk of LTBI reactivation, should also be considered.

## Figures and Tables

**Table 1:** Clinical characteristics of case reports of peritoneal tuberculosis in IBD patients under Infliximab despite a negative screening of LTBI.

Reference	Age, Sex	IBD	LTBI Screening Test	TB Location	IS at Screening	Time of Anti-TNF before TB (we)	IS at TB Diagnosis	Detection of TB
*U. Bonse-Geuking and M. Kraus* [25]	64, male	CD	TST (NEG.)	Peritoneal	Not specified	20	IFX + AZA	*M. tuberculosis* DNA in the peritoneal specimen, which was obtained via laparoscopy
*Y. K. Jun et al.,* [26]	27, female	UC	QFT-G (NEG.)	Disseminated (lung, peritoneal, lymph nodes)	Corticosteroids	28	IFX	*M. tuberculosis* culture that was resistant to rifampicin. Positive in lymph node
*A. Jauregui-Amezaga et al.,* [14]	38, female	CD	TST (NEG.)	Peritoneal	AZA	10	IFX + AZA	No microbiological detection. High levels of ADA in ascitic fluid.

UC: ulcerative colitis; CD: Crohn’s disease. we: weeks. TST: tuberculin skin test. NEG.: negative. QFT-G: Quantiferon Gold test. IS: immunosuppresion; AZA: azathioprine. IFX: infliximab. ADA: adenosine deaminase.

## Data Availability

Patient’s information are available anonymously upon request to the corresponding author.
